# A Wear Debris Segmentation Method for Direct Reflection Online Visual Ferrography †

**DOI:** 10.3390/s19030723

**Published:** 2019-02-11

**Authors:** Song Feng, Guang Qiu, Jiufei Luo, Leng Han, Junhong Mao, Yi Zhang

**Affiliations:** 1 School of Advanced Manufacture, Chongqing University of Posts and Telecommunications, Chongqing 400065, China; fengsong@cqupt.edu.cn (S.F.); s172131016@stu.cqupt.edu.cn (G.Q.); luojf@cqupt.edu.cn (J.L.); hanleng@cqupt.edu.cn (L.H.); 2 Theory of Lubrication and Bearing Institute, Key Laboratory of Education Ministry for Modern Design & Rotor-Bearing Systems, Xi’an Jiaotong University, Xi’an 710049, China; jhmao@mail.xjtu.edu.cn

**Keywords:** ferrography, classification of contours, segmentation of wear debris

## Abstract

Wear debris in lube oil was observed using a direct reflection online visual ferrograph (OLVF) to monitor the machine running condition and judge wear failure online. The existing research has mainly concentrated on extraction of wear debris concentration and size according to ferrograms under transmitted light. Reports on the segmentation algorithm of the wear debris ferrograms under reflected light are lacking. In this paper, a wear debris segmentation algorithm based on edge detection and contour classification is proposed. The optimal segmentation threshold is obtained by an adaptive canny algorithm, and the contour classification filling method is applied to overcome the problems of excessive brightness or darkness of some wear debris that is often neglected by traditional segmentation algorithms such as the Otsu and Kittler algorithms.

## 1. Introduction

During the operation of tribo-pairs, specific wear debris will be produced. The rate of debris generation, size distribution, shape and color are closely related to the wear state of tribo-pairs [1,2,3]. Analysis of wear debris in lube oil is of great significance for ensuring safe and efficient equipment operation [4]. Currently, many types of wear debris detection techniques have been developed [5,6].

Among the many technologies, machine vision-based methods can obtain more comprehensive wear debris characteristics, such as morphology, size and color, etc., which are hot spots in current research. Iwai et al. [7] developed an image-based wear debris dynamic counter, estimated the debris thickness by adjusting the imaging focal length to estimate the total wear amount, and verified the effectiveness on a friction and wear tester. Based on a similar method, Wu et al. [8,9,10,11] proposed a method of wear debris monitoring that realized a three-dimensional reconstruction of dynamic debris under low-information conditions, developed a classification algorithm for wear debris, and applied it to monitoring oxidative wear. Mabe et al. [12] designed a lensless debris online imaging device that integrates a microfluidic structure and an embedded image processor with an identification accuracy of 1 μm.

Ferrography is another important debris analysis technique. Compared with the wear debris analysis methods mentioned above, the biggest difference in ferrography is that a high-gradient strong magnetic field is used to deposit and separate wear debris from lube oil, which is then imaged to obtain a high-quality ferrogram [13]. Ferrography can evaluate the anti-wear properties of lube oil [14], wear type and wear intensity of tribo-pairs [15,16]. Ferrography has been widely used and has played a huge role in the analysis of mechanical system wear [17]. However, the traditional offline ferrography uses periodic manual sampling analysis, and there is information lag making it difficult to capture transient wear information; plus, it cannot fully characterize the behavior or natural degradation of a tribo-pair over time.

For real-time wear monitoring, Mao and Xie developed an online visual ferrograph (OLVF) using a combination of high gradient magnetic field deposition and image analysis techniques [18]. Wu et al. [19] proposed a wear debris segmentation algorithm for OLVF ferrograms using gray level and integrated morphological features and verified the effectiveness using gearbox oil samples. On this basis, OLVF is applied to automobile engine monitoring to capture abnormal wear, effectively avoiding serious faults [20,21]. Fan et al. [22] used OLVF to rapidly detect engines on the production line, capture the abnormal wear of the cylinder liner-piston pair, and isolate problem engines. Feng et al. [23] applied OLVF to monitor the gearbox wear process during the whole life cycle and established a model for the change in debris concentration to quantitatively analyze the gear wear process. The above studies are based on OLVF ferrograms under transmitted light conditions. Because of the deficiencies of the OLVF optical system, the topography and color characteristics of wear debris cannot be reliably obtained and oil transparency has a significant influence on image quality. When the lubricant ages and becomes black, it is impossible to effectively image wear debris.

In order to improve image quality, Li et al. [24] improved the optical imaging system of OLVF and designed a circular LED reflecting light element to achieve high brightness and uniform radiation. Then, a new mathematical model was proposed to analyze the illuminance uniformity of the improved OLVF CMOS image plane. The results show that the non-uniformity of the imaging plane is less than 5.6% [25]. Thereby, OLVF ferrograms are acquired under reflective conditions making it possible to extract surface morphology and color characteristics of wear debris. Further, Li et al. [26] proposed a new imaging structure that reflected wear debris under black oil conditions and expanded the application range for OLVF.

Existing wear debris extraction studies are mostly directed to transmission OLVF ferrograms, and few are directed to reflected light OLVF ferrograms. The difference between transmission and reflection ferrograms is huge. Unlike transmission ferrograms, reflection ferrogram information is more complicated, making it more difficult to extract debris features [27]. Wear debris segmentation is a prerequisite for extracting features such as color and morphology of wear debris, but there is currently no method for segmenting wear debris from reflected light OLVF ferrograms. For offline ferrograms, a variety of segmentation and debris feature algorithms have been proposed [28,29,30,31] that cannot be directly used for segmenting reflected light OLVF ferrograms because the different lighting conditions between OLVF and offline ferrographs lead to different wear debris appearance. Additionally, compared to offline ferrograms, there is often strong interference and relatively low contrast in OLVF ferrograms, and the time effectiveness of the image processing algorithm is relatively high.

In this paper, a method for wear debris segmentation based on edge detection and contour classification is proposed. First, a background subtraction method is used to simply distinguish the wear debris in the image, and then the morphological black hat operation is used to enhance the portion of the wear debris region that is close to the background color, and the bilateral filtering method is used to denoise the debris image. Next, the adaptive canny algorithm is used for edge detection to obtain wear debris contour information. Then, the inner contour is used to identify the enclosed area as the wear debris or the background by using a histogram similarity measure method. Finally, the classified wear debris contours are respectively determined. Filling is performed to achieve OLVF wear debris segmentation under reflected light conditions.

## 2. Methods

**Figure 1:**
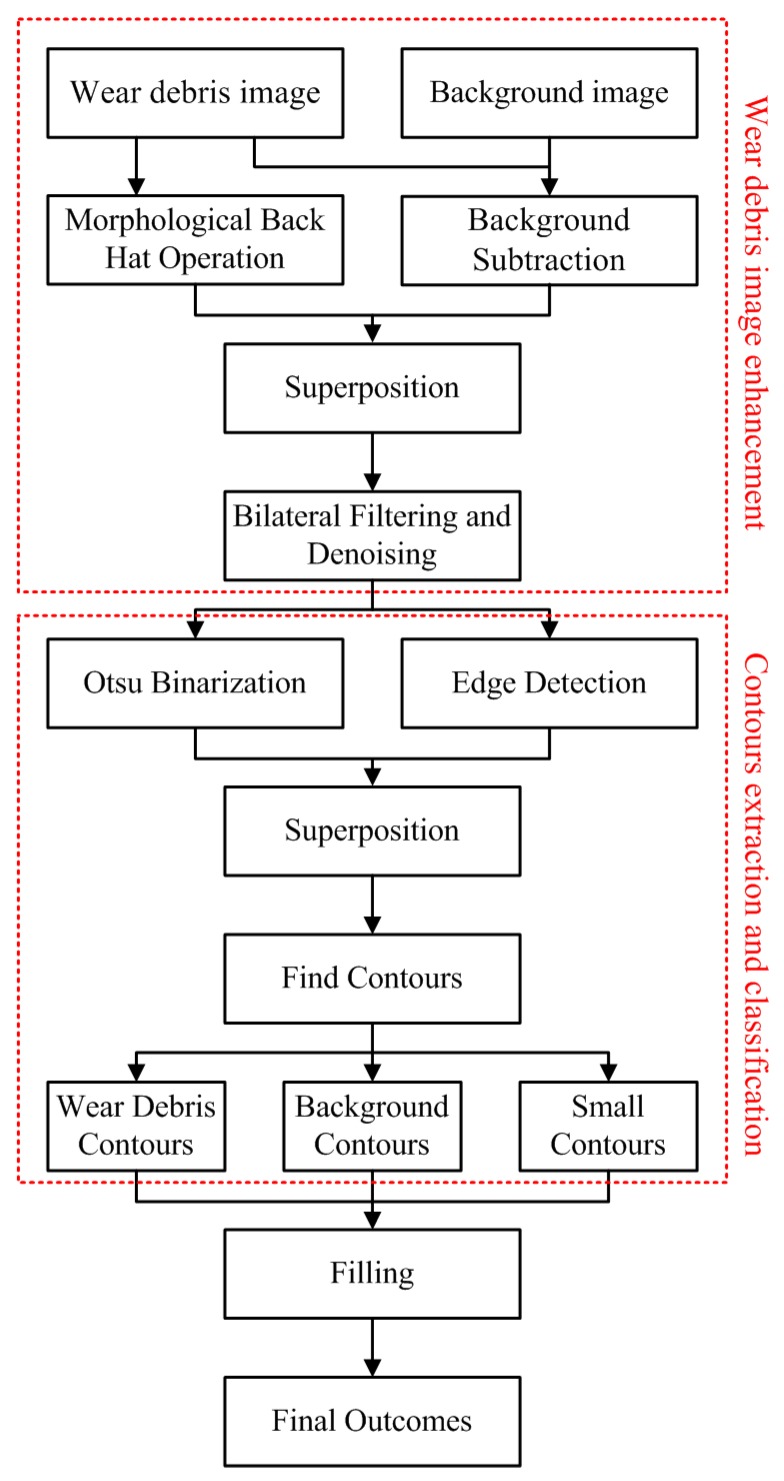
Proposed wear debris segmentation method.

In this paper, the wear debris was segmented using edge detection and contour classification methods to obtain a segmentation image for the extraction of subsequent wear debris features. First, background subtraction was performed to obtain a preliminary wear debris image. Second, the preliminary image was superimposed on the wear debris image treated by the morphological black hat operation to enhance parts of the wear debris region similar to the background image. Third, the enhanced debris image is subjected to denoising using a bilateral filtering method, and then the enhanced debris image was treated by Otsu binarization and edge detection before being superimposed. In addition, the contour classification method was adopted to perform contour differentiation for the images superimposed in the previous step, followed by wear debris filling and background filling, resulting in final binarized wear debris images. The conceptual diagram of the method is shown in Figure 1, and more detailed descriptions are shown below.

### 2.1. Wear Debris Image

**Figure 2:**
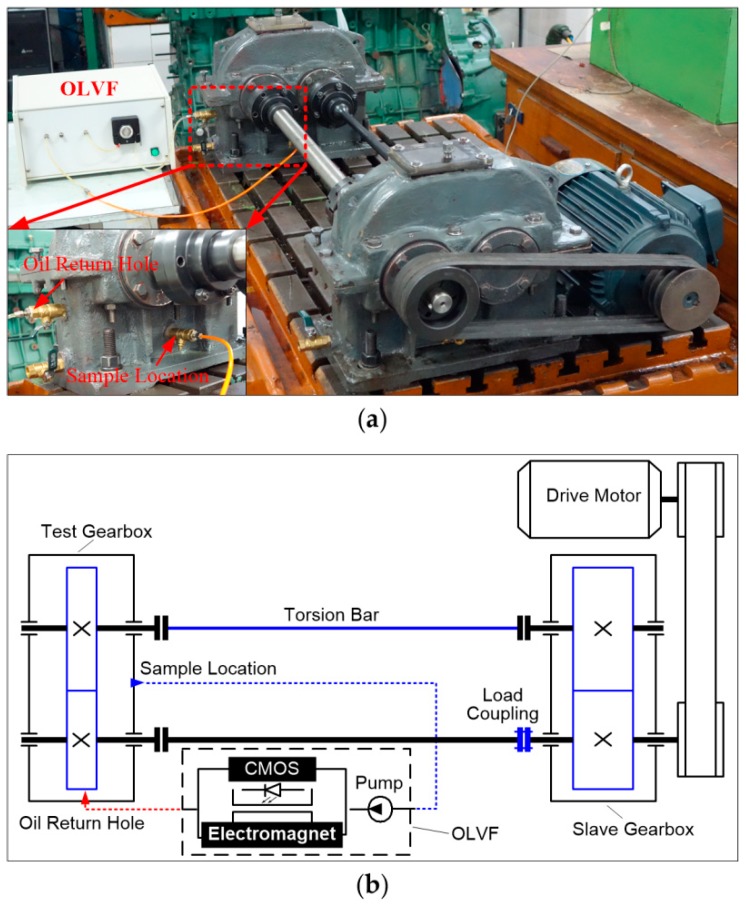
The back-to-back gearbox rig equipped with an online visual ferrograph (OLVF): (**a**) photograph; (**b**) schematic.

As shown in Figure 2, an OLVF is connected to a back-to-back gear rig. The in-used lube oil was drawn from the gearbox into the OLVF by a peristaltic pump and then drained back into the gearbox. The gear rig consisted of two oil-bath lubricated spur gearboxes. A torsion bar was twisted by relative rotation between two flanges of the load coupling to apply torque on the test gearbox. The gearbox rig was driven by an AC three-phase electrical motor (3 kW) at 1450 rpm. Test gear parameters are listed in Table 1. The lubricant was L-AN68 40# machine oil. Additionally, a computer communicated with the OLVF micro-control unit (MCU) to forward the sampling parameters, including the deposition time (30 s), sampling period (2 min), magneto-motive force (800 AN), flow rate of the rinse channel (10 mL/min), and flow rate of deposited debris (4 mL/min). The wear debris is separated from the lube oil in a high gradient magnetic field, and the reflected light OLVF ferrograms were obtained by an 8-bit CMOS camera (OV7725).

**Figure 3:**
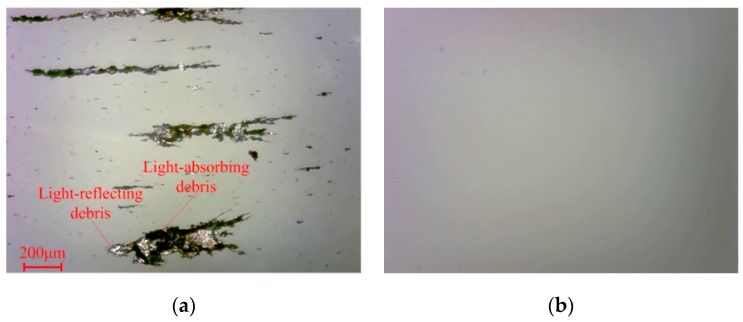
Typical OLVF ferrogram under reflected light: (**a**) wear debris image; (**b**) image background.

As seen from the typical wear debris ferrogram in Figure 3, when OLVF is imaging wear debris, the debris is wrapped in lube oil. Consequently, the wear debris image suffers from considerable interference caused by light, the oil transparency, etc. In the OLVF ferrogram, there is either darker or brighter wear debris influencing the contrast differences between the wear debris and background, leading to missing some when segmenting wear debris and mistakenly recognizing the partial interference shadow as wear debris. Unfortunately, the defects mentioned above cause large errors when extracting wear debris features.

As seen in Figure 3a, the initial wear debris images contain considerable noise, preventing complete distinguishing of wear debris from the background. Therefore, it was necessary to enhance the initial wear debris image to remove noise.

### 2.2. Wear Debris Image Enhancement

**Figure 4:**
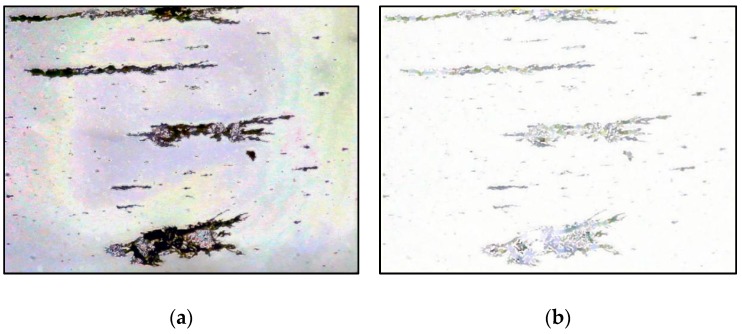

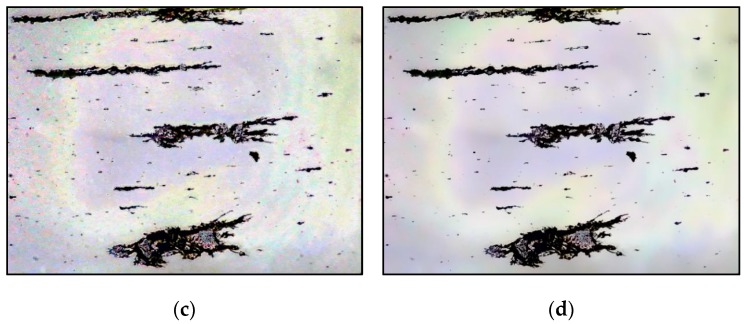
Wear debris image enhancement: (**a**) background subtraction; (**b**) black hat operation; (**c**) superimposed wear debris image; (**d**) bilateral filtering.

Background subtraction was performed first. The background image was subtracted from the wear debris image to obtain a preliminary processed wear debris image. The result is shown in Figure 4a. It can be seen that the noise in the wear debris caused by uneven illumination is passed on to the image obtained after background subtraction. In addition, wear debris regions with similar color to the background has a low contrast ratio and is difficult to identify.

Therefore, the original wear debris image was processed using the morphological black hat operation that the closed operation image subtracts the original image. Its function is to highlight areas darker than the area around the contour of the original image, as shown in Figure 4b. By superimposing Figure 4a,b, the wear debris regions, similar color to the background, are strengthened. The superimposed image is shown in Figure 4c. Obviously, there is still noise in the superimposed wear debris image passed from the original wear debris image. Thus, the bilateral filtering method was used to denoise it. The result is shown in Figure 4d.

### 2.3. Initial Segmentation of Wear Debris Edge Detection Based on an Adaptive Canny Operator

The enhanced wear debris image applied edge detection based on the self-adaptive canny operator. The main processes included applying a Gaussian function to the filter, calculating the gradient and amplitude, and constructing a gradient histogram. The first derivative of the two-dimensional Gaussian function was used to low-pass filter the wear debris. Let the two-dimensional Gaussian function be:

(1) $$G\left(x,y\right)=\frac{1}{2\pi \sigma^{2}}\exp\left(-\frac{x^{2}+y^{2}}{2\sigma^{2}}\right)$$

The gradient vector is:

(2) $$\nabla G=\left[\frac{\partial G}{\partial x},\frac{\partial G}{\partial y}\right]$$

where:

(3) $$\frac{\partial G}{\partial x}=kx\;\exp\left(-\frac{x^{2}}{2\sigma^{2}}\right)\exp\left(-\frac{y^{2}}{2\sigma^{2}}\right)$$

(4) $$\frac{\partial G}{\partial y}=ky\;\exp\left(-\frac{x^{2}}{2\sigma^{2}}\right)\exp\left(-\frac{y^{2}}{2\sigma^{2}}\right)$$

where *k* is a constant and σ  is a Gaussian filter parameter that controls the smoothness of the image.

For the enhanced wear debris image, the image gradients in the x and y directions were solved and denoted as $p_{x}\left(i,j\right)$ and $p_{y}\left(i,j\right)$, respectively. Then, the gradient magnitude of wear debris is:

(5) $$M\left(i,j\right)=\sqrt{p_{x}{}^{2}\left(i,j\right)+p_{y}{}^{2}\left(i,j\right)}$$

Based on the gradient magnitude obtained from the above formula, a wear debris image gradient histogram was constructed.

To determine the optimal segmentation threshold for the high-low gradient region in the gradient histogram, the self-adaptive canny algorithm steps are as follows:

1. Select an initial estimate for threshold *T*_0_;
2. The self-adaptive canny algorithm steps are as follows. The image is segmented using a threshold *T*_0_ in which case two sets of pixels are generated: Image *G*_1_ is constituted by all pixels with a gray value greater than or equal to *T*_0_, and image *G*_2_ is constituted by all pixels with a gray value smaller than *T*_0_;
3. Calculate the average gray values *μ*_1_ and *μ*_2_ in the range of *G*_1_ and *G*_2_;
4. Calculate new thresholds *T* = (*μ*_1_ + *μ*_2_)/2;
5. Repeat steps 2 through 4 until the threshold changes in successive iterations are smaller than the pre-specified parameters *T*_0_.

**Figure 5:**
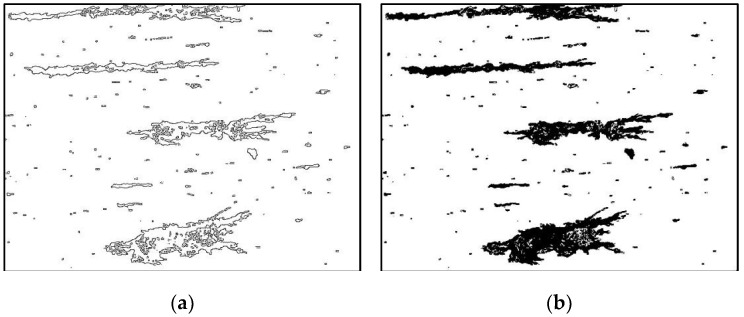
Superimposed wear debris image: (**a**) Otsu method; (**b**) superposition of the wear debris image after binarization and treatment by the adaptive canny algorithm.

The optimal segmentation threshold for the high-low gradient region in the gradient histogram was obtained, and image edge tracking is performed, thereby realizing edge detection. The results are shown in Figure 5a.

Figure 5b is the result of superposition of the wear debris image after binarization and the wear debris image treated with the adaptive canny algorithm. From the binarized wear debris image, it can be seen that the contour boundary line is not continuous, whereas in the wear debris image processed by the adaptive canny algorithm the contour boundary line is clear and continuous. Through superposition of the two images, the contours of the binarized wear debris image are more continuous and accurate.

### 2.4. Contour Classification

As seen from the segmented image in Figure 5b, contour lines of many wear debris regions are not continuous, holes can be observed, and large errors exist in segmentation. By expanding the boundary chain code of the preliminary segmented wear debris image, wear debris shape parameters were extracted, so that the identified contour could be divided into large and small contours in terms of area and divided into contour and outer contours in terms of location. To further distinguish whether the area within the contour was wear debris or image background, we calculated the histograms of the wear debris and background images within the inner contour range, as well as the Bhattacharyya distance between the two histograms. Then, the similarity between the two histograms was determined.

Statistically, the Bhattacharyya distance measures the similarity of two probability distributions. It can be used to calculate the relative closeness of the two samples being considered and can be applied in target detection and tracking.

For probability distributions p  and q over the same domain X, the Bhattacharyya distance is defined as:

(6) DB(p,q)=−ln(BC(p,q)),         0≤DB<+∞

where BC(p,q) is the Bhattacharyya coefficient, and the calculation formula is:

(7) $$BC\left(p,q\right)=\underset{x\in X}{\sum }\sqrt{p\left(x\right)q\left(x\right)},\;\;\;\;\;\;\;\;0\leq BC\leq 1$$

Equation (8) is the discrete distribution function. The continuous distribution function is defined as:

(8) $$BC\left(p,q\right)=\int \sqrt{p\left(x\right)q\left(x\right)},\;\;\;\;\;\;\;\;0\leq BC\leq 1$$

**Figure 6:**
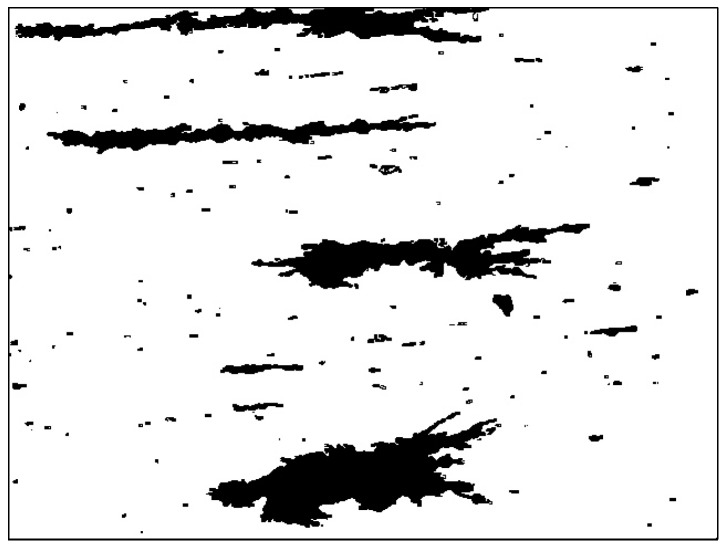
Final binarized wear debris image.

The Bhattacharyya distance was used to calculate the similarity between the target and probabilistic models for the initial image. The higher the similarity, the better is the representation of the target in the image area. Thus, the contour area could be distinguished as wear debris or background. The contour area of the debris was filled to finally obtain the binary wear debris image. The effect diagram is shown in Figure 6. The hole in the wear debris area was removed completely, and the contour was clear and coherent. Better wear debris segmentation was obtained.

## 3. Results and Discussions

**Figure 7:**
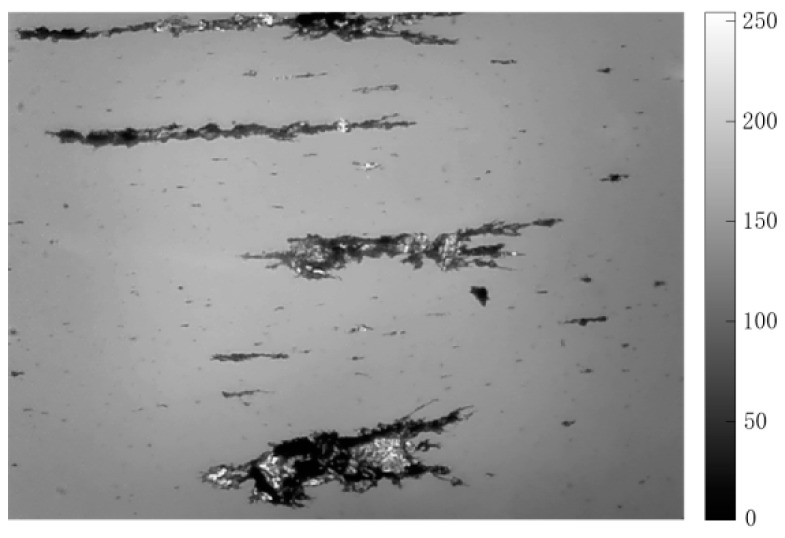
Topography of the grayscale ferrogram.

**Figure 8:**
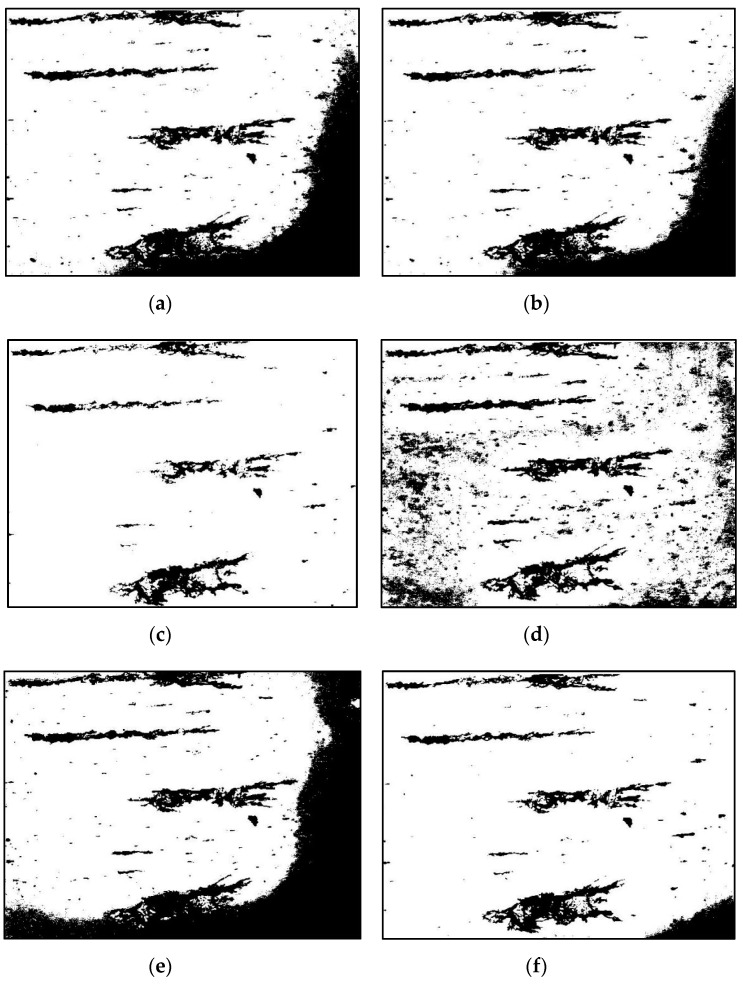
Images produced by different binarization methods: (**a**) Otsu method; (**b**) iterative method; (**c**) Kittler method; (**d**) Niblack method; (**e**) cyclic threshold method; and (**f**) fixed threshold method.

The topography of the grayscale ferrogram is illustrated in Figure 7. The gray level of the background image is about 150, and the grayscale range of wear debris is large. Light-absorbing debris is darker than the background, whereas reflective light debris is the opposite. Consequently, some threshold segmentation results are erroneous. Figure 8a depicts a binarized image using the Otsu algorithm. The global threshold selected on the histogram characteristics cannot effectively identify debris with a large grayscale. Because the gray value distribution of the background is not uniform, the selected global threshold mistakenly identifies part of the background area as wear debris, making it difficult for the Otsu method to effectively segment wear debris under reflected light, and the corresponding area appears to be fragmented. The image was divided by an approximate threshold to find the average gray value, and then a new threshold was set until that value does not change in the two parts. Using this process, a binarized image produced with the iterative algorithm is shown in Figure 8b. The advantage of this method is that the segmentation effect for the region with higher discrimination between the debris and the background is obvious, and the smaller debris region has better segmentation. However, areas with dark backgrounds are identified as wear debris, and wear debris under reflected light cannot be effectively segmented.

The binarized image shown in Figure 8c produced using the Kittler algorithm could obtain the optimal segmentation threshold based on the minimum error of the wear debris and the background image. Therefore, the method has an obvious effect on image segmentation with a large difference between the wear debris and background variance, and the influence of uneven illumination is suppressed to some extent, but a considerable amount of small wear debris with low contrast is filtered out. In addition, light-reflecting wear debris is not effectively identified.

As for Figure 8d, the binary wear debris image produced using the Niblack algorithm obtained the binarization threshold calculated by the mean and standard deviation of the pixel point gray values near a certain pixel and its neighborhood, thereby performing local area binarization. When the selected areas all contain debris, segmentation is more prominent, but if the selected regions are background points, the algorithm treats the points with higher gray values as wear debris, leading to the introduction of pseudo noise.

In Figure 8e, the grayscale image is converted from wear debris and then divided into two parts to obtain the average gray value, and the average threshold for segmentation is set to produce the binary image using the loop threshold algorithm. This method is similar to the iterative method. It has good segmentation of the wear debris in a small area, but it is significantly affected by the illumination factor, and identifying and segmenting the wear debris under the reflected light is difficult. Figure 8f is the image obtained by binarization of a fixed threshold method, and the segmentation threshold can be adjusted at any time to optimize the image segmentation. However, the grayscale of the debris is large, and the background grayscale distribution is not uniform. The selected threshold always loses some of the debris characteristics, or some of the darker light regions may be mistakenly identified as debris.

**Figure 9:**
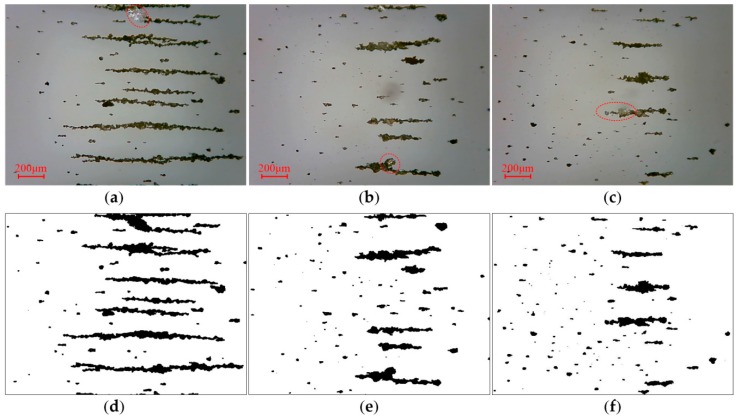
Segmentation of ferrograms with small wear debris: (**a**–**c**) reflected light OLVF ferrograms; (**d**–**f**) binarization images.

**Figure 10:**
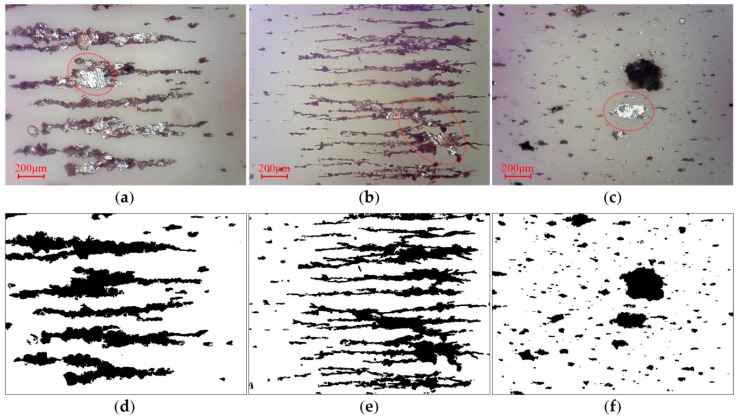
Segmentation of ferrograms with large wear debris: (**a**–**c**) reflected light OLVF ferrograms; (**d**–**f**) binarization images.

To further verify the effectiveness of the algorithm, experiments were performed on sets of small and large debris ferrograms. The red dotted circles in the pictures indicate reflective regions of wear debris. For the small debris ferrograms, the algorithm can obtain better segmentation. Figure 9 and Figure 10a,b show that in large debris ferrograms, wear debris adheres to itself to easily form a background region surrounded by the inner contour, and the identification of these regions is key to wear debris segmentation. As shown in Figure 10d,e the algorithm can better identify these areas. For small and large wear debris ferrograms, the algorithm can get better segmentation results. Compared with the above methods, the wear debris segmentation method proposed in this paper removes the noise caused by illumination and other factors and fills the binary image using the contour filling method and ensuring wear debris capture. Our method simultaneously has better segmentation.

## 4. Conclusions

Under reflected light, the OLVF particle ferrogram suffers from interference that negatively impacts wear debris segmentation. Some wear debris is darker than the background and some is brighter, which may lead to missing wear debris of similar color to the background when using the traditional segmentation method. In this study, a wear debris segmentation algorithm based on edge detection and contour classification was proposed. First, the morphological black hat operation was performed to highlight the wear debris of similar color to the background. The wear debris image was enhanced by bilateral filtering, and the image was subjected to preliminary segmentation by the adaptive canny operator. Then, the contour of the preliminarily segmented image was searched. For the inner contour, the histogram similarity measurement method was used to identify whether the area enclosed by the inner contour was wear debris or background. Finally, the contours of classified wear debris were filled to achieve OLVF wear debris segmentation under reflected light. Compared with the traditional segmentation method. The proposed method effectively recognizes both light-reflecting and light-absorbing debris regions, and a more accurate wear debris segmentation is obtained, which is helpful for improving morphological characteristics extraction and pattern recognition of wear debris.

## Figures and Tables

**Table 1:** Details of test gears.

Parameter	Pinion	Wheel
Number of teeth	17	24
Module (mm)	6	
Center distance (mm)	125	
Pressure angle (°)	20	
Addendum modification	+0.282	+0.0707
Face width (mm)	10	
Roughness Ra (μm)	2–3	
Hardness (HRC)	40–45	
Material	45#	
